# Case report: Fatal infantile hypertonic myofibrillar myopathy with compound heterozygous mutations in the *CRYAB* gene

**DOI:** 10.3389/fped.2022.993165

**Published:** 2023-01-16

**Authors:** Shan-shan Zhang, Li-niu Gu, Teng Zhang, Lu Xu, Xiang Wei, Su-hong Chen, Su-jie Shi, Da-quan Sun, Shao-hong Zhou, Qian-ye Zhao

**Affiliations:** ^1^ Pediatric Respiratory Ward I, Lianyungang Maternal and Children's Hospital, Lianyungang, China; ^2^ Department of Immunization Planning, Lianyungang Center for Disease Control and revention, Lianyungang, China; ^3^ Department of Science & Education, Lianyungang Maternal and Children's Hospital, Lianyungang, China; ^4^ Department of Neonatology, Lianyungang Maternal and Children's Hospital, Lianyungang, China

**Keywords:** CRYAB, fatal infantile hypertonic myofibrillar myopathy, gene, mutation, whole exome sequencing

## Abstract

**Background:**

Fatal infantile hypertonic myofibrillar myopathy (FIHMM) is an autosomal recessive hereditary disease characterized by amyotrophy, progressive flexion contracture and ankylosis of the trunk and limb muscles, apnea and respiratory failure, and increased creatine phosphate levels. It is caused by mutations in the *CRYAB* gene, and only around 18 cases including genetic mutations have been reported worldwide. All patients with FIHMM develop respiratory distress, progressive stiffness of the limbs, and have a poor prognosis. However, no effective treatment for *CRYAB*-associated respiratory failure has been reported. Here, we report a case of FIHMM with a novel heterozygous missense mutation.

**Case Presentation:**

A 2-year-old female developed scoliosis of the lumbar spine and restrictive ventilatory dysfunction in infancy. She was admitted to the hospital with labored breathing on the third day after the second injection of inactivated poliomyelitis vaccine. Acute respiratory failure, pneumothorax, and cardiac arrest arose in the patient during hospitalization, and progressive stiffness of the trunk and limb muscles appeared, accompanied by obvious abdominal distension and an increase in phosphocreatine kinase levels. Screenings for genetic metabolic diseases in the blood and urine were normal. Electromyography revealed mild myogenic damage. A muscle biopsy indicated the accumulation of desmin, *α*-crystallin, and myotilin in the musculus biceps brachii, and dense granules were observed in muscle fibers using electron microscopy. Mutation analysis of *CRYAB* revealed a novel heterozygous missense mutation in the proband, *c.302A > C* (*p.His101Pro*) and *c.3G > A* (*p.Met1Ile*), which inherited from her asymptomatic, heterozygous carrier parents, respectively. The proband was finally diagnosed as FIHMM. One month after the FIHMM diagnosis, the child died of respiratory failure.

**Conclusion:**

We report a case of FIHMM with a novel heterozygous missense mutation of *CRYAB*. This finding might improve our understanding of FIHMM and highlight a novel mutation in the Chinese population.

## Introduction

Fatal infantile hypertonic myofibrillar myopathy (FIHMM) is an autosomal recessive hereditary disease caused by mutations in the *CRYAB* gene. Symptoms such as weak crying, sleep apnea, recurrent respiratory tract infection, and progressive stiffness of the trunk, abdomen, and limbs are observed during the neonatal period or in early infancy (1–4). All patients suffer from respiratory failure; thus, the disease has a poor prognosis. To date, no effective treatment has been reported for FIHMM. Here, we describe a case of FIHMM in which a novel compound heterozygous missense mutation of *CRYAB* was found.

## Case presentation

### Ethical compliance

The proband's parents provided written informed consent to participate in this study in compliance with the Declaration of Helsinki, and the Institutional Review Board at Lianyungang Maternal and Child Health Hospital approved this study (project no. LYG-MEP2021004).

A 2-year-old female was hospitalized for respiratory distress on the third day after the second injection of inactivated poliomyelitis vaccine. Oxygen saturation fluctuated from 68% to 92% in ambient air. The child showed no fever, no coughing and wheezing, no vomiting and diarrhea at early onset. She had been admitted to our hospital previously for premature delivery (gestational age: 29 weeks and 4 days), neonatal shock, intrahepatic cholestasis, bronchopulmonary dysplasia, and extrauterine growth retardation for more than 70 days, and she had suffered from severe bronchopneumonia, pneumothorax (twice), scoliosis of the lumbar spine, and restrictive ventilatory dysfunction at 1 year old. She was G_3_P_1_ and delivered with a birth weight of 1.2 kg. Her mother had aborted twice previously for embryo arrested development. Her parents are both healthy, and there was no family history of similar symptoms.

Admission Physical Exam: T.37 °C, P.160/min, RR.50/min, Wt.10 kg, BP.90/60 mmHg, SaO_2_.70%. The patient was malnourished, looked mentally fatigued, and suffered from dyspnea. The pharynx was slightly congested, and the neck was soft. Inspiratory three-concave signs were positive. Bilateral respiratory motion and tactile fremitus were symmetric bilaterally. There were some rough and middle moist rales predominantly in the pulmonary bilateral middle-inferior field according to chest auscultation, but no wheezing or pleural friction sounds were heard. Her heart rate was 160 beats per minute, and her cardiac rhythm was regular. In the precordial area, a 2/6 systolic blowing cardiac murmur was detected. The abdomen was distended. No hepatosplenomegaly was found. There was serious kyphosis of the lumbar spine. The four limbs could be moved freely. No abnormalities were found in a nervous system examination.

**Figure 1:**
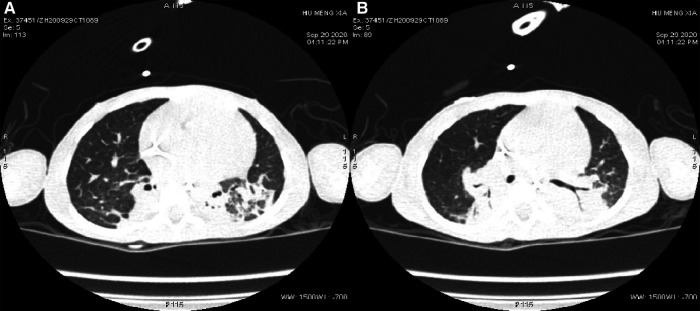
Changes in pulmonary imaging. (**A**) Consolidation of the bilateral lower lobe; (**B**) atelectasis of the posterior basal segment of the right lung.

**Figure 2:**
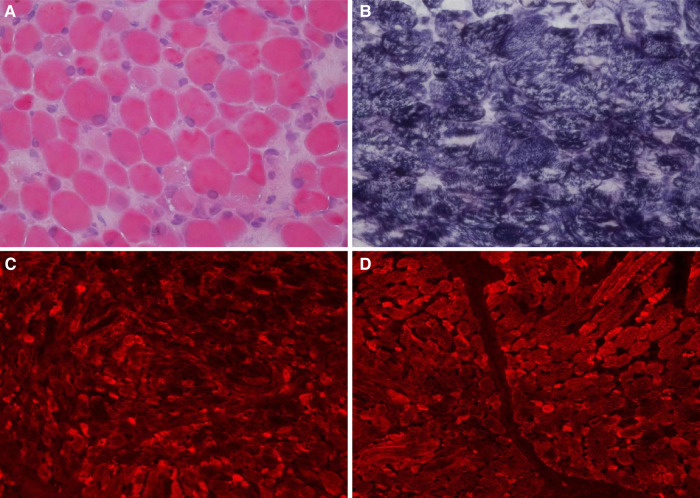
Histochemical and immunohistochemical staining of muscle biopsies (×200). Following HE staining (**A**), different sizes of muscle fibers were observed, and degeneration, necrosis, and regeneration of the muscle fibers was evident as well as the deposition of amorphous materials. Some muscle fiber staining was significantly darker than the surrounding myofibrils according to modified Gömöri trichrome staining (**B**). Fluorescence antibody staining of desmin, *α*-crystallin (**C**), and myotilin (**D**) revealed high expression levels of these proteins in the sediment.

Auxiliary Examinations: No abnormalities were found in all tests, including routine blood, high-sensitivity C-reactive protein, and routine urine and stool tests. The results of PCR tests for nine respiratory viruses, EB virus, thirteen respiratory bacteria, *Mycoplasma pneumoniae*, and *Chlamydia pneumoniae* were all negative. Five items of coagulation, renal function, troponin, and hematuria tandem mass spectra were all in the normal range. Procalcitonin levels fluctuated from 1.651 to 7.480 ng/ml. Creatine phosphate kinase levels kept increasing. Arterial blood gas analysis showed an increase in PaCO_2_ under the support of a ventilator. Bronchoalveolar lavage fluid and sputum cultures indicated the presence of *Candida tropicalis*, *Burkholderia cepacia*, and *Acinetobacter baumannii*. Cardiac color Doppler ultrasound showed the presence of the foramen ovale, aortic regurgitation (mild to moderate), slight thickening of the ventricular septum and left ventricular posterior wall, and normal cardiac function. A chest and abdominal CT scan indicated the presence of bilateral pneumonia (Figure 1) and pleural effusion, enlarged liver volume, a small effusion around the liver, a strip-shaped high-density focus in the gallbladder (nature to be determined), thickened bilateral abdominal wall muscle tissue, and atrophic bilateral erector spinal muscles. A spinal CT scan showed scoliosis of the lumbar spine and no bone destruction in each vertebral body. There were no spontaneous potentials in acupuncture electromyogram of anterior tibialis, medial peroneal muscles, vastus medialis, medial femoral, the first dorsal interossei, musculus biceps brachii and musculus triceps brachii. The conductive velocity and amplitude of sensory and motor nerve were at the normal range, and consequently, mild myogenic damage was deduced. Histochemical staining (H&E, Gomori Trichrome, ORO and PAS), enzyme histochemistry and special staining (NADH-TR, SDH, COX, SDH / COX, ATPase, ACP, AMP, NSE, phosphorylase and PFK), immunohistochemistry staining (Alexa Fluor or FITC labelled Desmin, *α*-Crystallin, Myotilin, titin, dystrophin, *α*, *β*, *γ*, *δ*-Sarcoglycan, Dysferlin, Laminin*α*2), and monoclonal antibody staining of Collagen VI were conducted in muscle biopsy. The Pathological results showed the accumulation of desmin, *α*-crystallin, and myotilin in the musculus biceps brachii, and dense granules were observed in muscle fibers using electron microscopy, which demonstrated myogenic fibromyopathy (Figure 2).

### Diagnostic assessment

Based on the above-described clinical findings, we inferred a possible diagnosis of hereditary myopathy. To confirm this diagnosis, whole exome sequencing (WES), conducted at the Wuhan Kangshengda Medical Research Institute, was used by a high-throughput analysis technique. The data were filtered to generate “clean reads” by removing adapters and low-quality reads (Q20). Sequencing reads were mapped to the reference human genome version hg19 (2009–02 release, http://genome.ucsc.edu/). Nucleotide changes observed in the aligned reads were called and reviewed using NextGENe software (SoftGenetics, State College, PA). In addition to the detection of deleterious mutations and novel single-nucleotide variants, sequence variants were annotated using population and literature databases including dbSNP, TGP, Clinvar, and HGMD. All variants were elucidated according to ACMG standards and categorized as pathogenic, likely pathogenic, uncertain significant,, likely benign and benign (5). Possible pathogenicity was predicted according to the online tools of PolyPhen-2, and MutationTaster. Moreover, reanalysis of the WES results were conducted on the patient and her parents.

**Figure 3:**
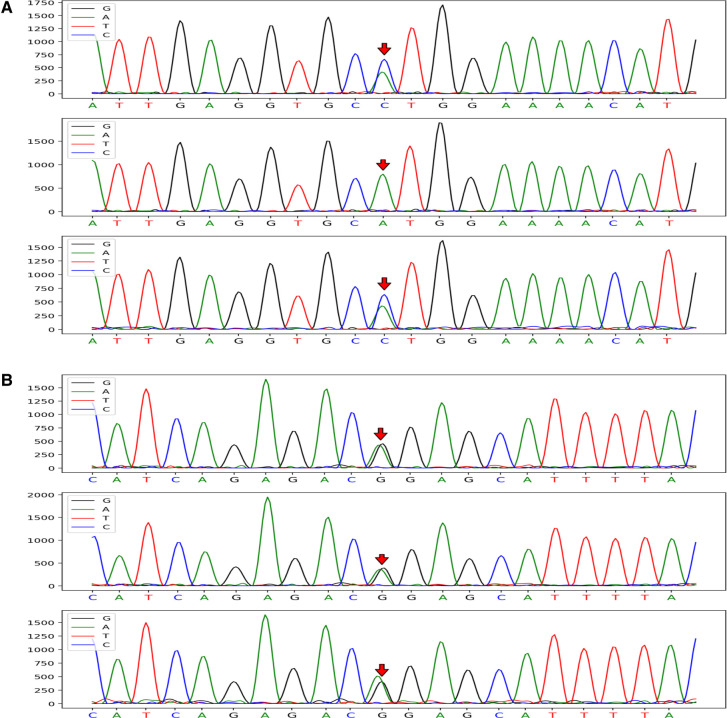
*CRYAB* gene sequences of the proband and her parents (mutation sites are marked with arrows). The *CRYAB* c.302A > C (p.H101P) mutation was located in the region of exon 3 of chromosome 11 and resulted in the mutation of the NO. 302 nucleotide from adenine to cytosine (**A**). The proband with FIHMM and her mother carried the same gene mutation; however, her father's gene locus was normal. The *CRYAB* 3G > A (p.M1I) mutation was located in the region of exon 2 of chromosome 11 and caused the mutation of a synthetic amino acid from methionine to isoleucine (**B**). The mutation carried by the proband was inherited from her unaffected father, whereas her mother's gene locus was normal.

**Figure 4:**
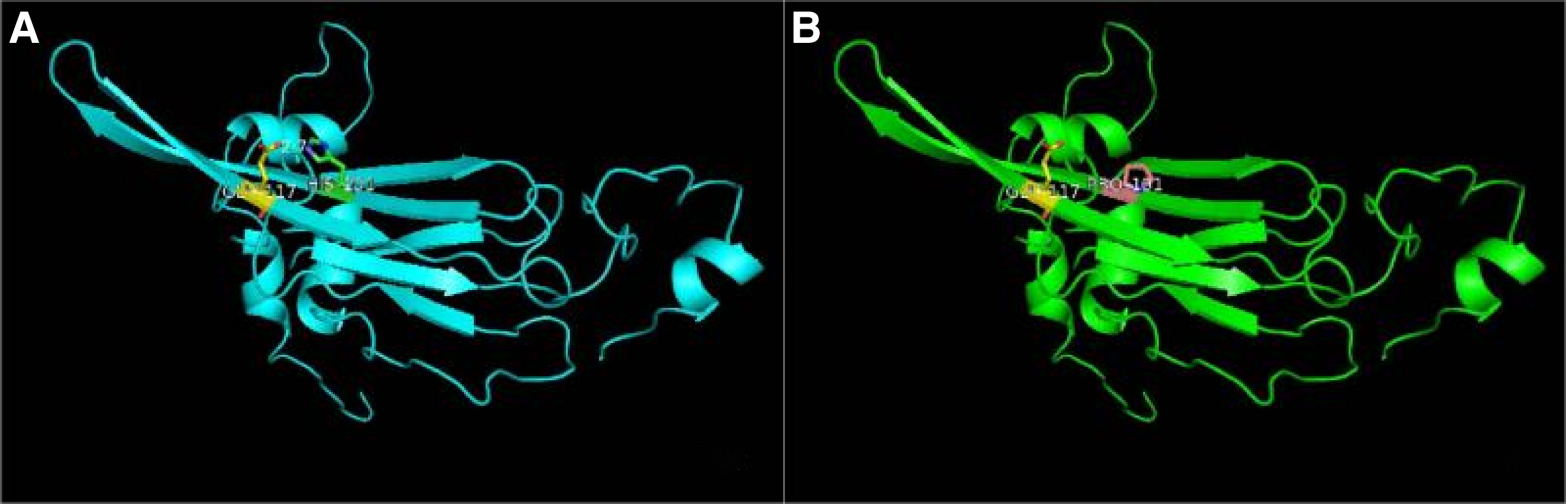
Effect of the *CRYAB* c.302A > C (p.H101P) mutation on the three-dimensional structure of the protein predicted by functional model software. (**A**) Wide type and (**B**) mutant type. A hydrogen bond was absent in the mutation site of *α*B-crystallin.

CRYAB gene complex with heterozygous mutations was found in the proband (GeneBank reference sequence: NM_001885). The variant, *CRYAB* (NM_001885): *c.302A > C* (*p.His101Pro*), was located in the region of exon 3 of *CRYAB*, in which the NO.302 nucleotide was mutated from adenine to cytosine (*c.302A* > *C*), leading to a mutation of the NO.101 amino acid from histidine to proline (Figure 3A), which was inherited from currently asymptomatic, heterozygous carrier mother. According to *ACMG Standard and Guidelines for the Interpretation of Sequence Variants*, the mutation is uncertain significance with two moderate evidence of pathogenicity (PM1 and PM2), and a supporting evidences of pathogenicity (PP3). The variant was predicted to be “probably damaging” by PolyPhen2 with a score of 0.995 (sensitivity: 0.04, specificity: 0.98) (6) and “disease causing” by MutationTaster with a score of 1. The Swiss-Model computer model was used to predict the influence of this identified mutation on protein structure, with the analysis conducted by Hangzhou Waylan Biotechnology Co. The *CRYAB c.302A *> *C* (*p.His101Pro*) mutation affected the formation of a hydrogen bond (Figure 4), which influenced the three-dimensional structure of the CRYAB protein severely and caused functional defects, indicating the disease causing mutation.

The other variant, *CRYAB* (NM_001885): *c.3G *>* A* (*p.Met1Ile*) (provided in Data S.), inherited from her unaffected heterozygous carrier father, was located in the region of exon 2 of *CRYAB* (Figure 3B), in which the NO.3 nucleotide was mutated from guanine to adenine (*c.3G > A*), resulting in a mutation of the NO.1 amino acid from methionine to isoleucine. The mutation is clearly pathogenic with a very strong evidence of pathogenicity (PVS1) and a moderate evidence of pathogenicity (PM2) (detailed in Data S). According to the N-terminal rule (7), the *CRYAB c.3G > A* (*p.Met1Ile*) mutation shortens the half-life of the CRYAB protein, thereby decreasing its stability. Result showed the variant is probably damaging with a score of 0.999 (sensitivity: 0.14, specificity: 0.99) using PolyPhen-2 tool, and “disease causing” by MutationTaster with a score of 1, which suggests that there is a high probability that the *CRYAB c.3G > A* (*p.Met1Ile*) mutation may be the cause of FIHMM. The list of the variants left after final filtering step are provided in the Data S. However, it was not clear whether the two intrauterine fetal deaths suffered by the proband's mother were related to gene abnormality because WES screening was not performed on that occasion.

## Discussion

FIHMM was first reported among mid-western Canadian aboriginals in 1994 (2, 8) identified *CRYAB* as the gene responsible for this disease. *CRYAB* is located in chromosomal region 11q22.3∼q23, is composed of three exons and 175 nucleotides (9), and encodes *α*B-crystallin (10), also known as HSPB5, which is a member of the small heat-shock protein family and a molecular chaperone that interacts with desmin in the assembly of intermediate filaments. These act as chaperones to prevent protein aggregation (11), stabilize myofibrils, maintain muscle fiber elasticity, allow remodeling after repeated contraction (2, 12), inhibit the aggregation and precipitation of denatured proteins, and prevent loss of function under stress conditions (13). Due to the high expression of *α*B-crystallin in human type-I myofibers, *CRYAB* variants often cause selective muscle fibrosis atrophy of the trunk, spine, and limbs, such as in myofibrillar myopathy type II (14–16), dilated cardiomyopathy 1 type II (9), FIHMM (8), and multitype cataracts (17, 18). Among these diseases, FIHMM is an autosomal recessive hereditary disease that generally arises in the early period as a serious muscular atrophy condition (2, 4, 14), with clinical manifestations including progressive stiffness of the trunk and limb muscles, flexion contracture (19), ankylosis, faint crying, dysphagia and dyspnea. Immunohistochemistry staining of muscle biopsies showed accumulations of desmin, and dense granules in the myocytes using electron microscopy.

To date, *CRYAB*-related fatal infantile hypertonic MFM (OMIM 613869), that is FIHMM, was induced primarily by homozygous mutations in *CRYAB* gene. Two sporadic cases included *CRYAB c.3G *>* A (p.Met1Ile)* (3) and *CRYAB c.343delT (p.Ser115ProfsX14)* (19) mutations, respectively. Among the Canadian aboriginal population, the variant, *CRYAB c.60delC* (*p.Ser21AlafsX24*) homozygous mutation was described in 12 cases with FIHMM (2). Lu et al. (4) reported four Chinese cases with the homozygous mutation gene in *CRYAB c.3G > A* (*p.Met1Ile*), that results in the loss of methionine initiation and the absence of a protein, which was different with our report, a heterozygous missense mutation in *CRYAB gene, c.302A *>* C* (*p.His101Pro*) and *c.3G *>* A* (*p.Met1Ile*).

In our report, the heterozygous missense mutations in the *CRYAB* gene of the proband were inherited from her currently asymptomatic parents. The proband showed recurrent respiratory tract infection in infancy, and gradually developed the symptoms of erector spinae muscle atrophy and lumbar kyphosis deformity without bone destruction in the vertebra lumbalis after the age of 1 year. As the illness progressed, the proband presented with a stiffness of the trunk and limb muscles, abdominal distension, uncorrectable respiratory failure (type II), and increasing creatine phosphate kinase levels. Finally, the child died after artificial respiration support was ended. The clinical feature of this case was in accordance with *CRYAB c.3G > A* (*p.Met1Ile*) homozygous mutation related FIHMM phenotype reported in the literature (2, 8). Electromyography results of the proband were consistent with myogenic damage, and the muscle biopsy showed the accumulation of desmin, *α*-crystallin, myotilin and dense granules in the musculus biceps brachii. Meanwhile, immunohistochemistry staining of titin, dystrophin, *α*, *β*, *γ*, *δ*-Sarcoglycan, Dysferlin and Laminin*α*2 were all negative in muscle biceps. Except for *CRYAB*, Fifteen mutation genes associated with Myofibrillar myopathy (MFM) phenotype, including five classic genes *DES*, *ZASP*, *FLNC*, *BAG3*, *MYOT*, and other genes *FHL1*, *TTN*, *DNAJB6*, *PLEC*, *LMNA*, *ACTA1*, *HSPB8*, *KY*, *PYROXD1*, and *SQSTM* + *TIA1* (digenic) were not found in the results (20, 21). Meanwhile, FIHMM as a atypical MFM, manifest a progressive stiffness of the trunk and limb muscles. The mutation in DMPK associated with tonic muscular dystrophy was not identified. Therefore, according to the clincal phenotype, relevant pathological results, and combined with gene mutations in *CRYAB:c.302A *> *G* (*p.His101Pro*) and *c.3G *> *A* (*p.Me*t*1Ile*), the proband was finally diagnosed as FIHMM, which conformed to the law of recessive inheritance. The two variants were not the same allele. We speculate that there is a possible involvement of complex genetic background. It is hard to pinpoint. *CRYAB c.302A *> *G* (*p.His101Pro*) as a variant of minor pathogenicity might help the other variant with very strong pathogenicity, *CRYAB c.3G* > *A* (*p.Met1Ile*), produce pathogenic results. The relevant mechanism still needs further research.

In conclusion, this article reports one infant with FIHMM with a novel heterozygous variant in *CRYAB*. The report broadened genetic spectrum of the *CRYAB* mutations related FIHMM. This study may aid in diagnosis, carrier detection, and genetic counsel of *CRYAB*-related neonatal onset MFM.

## Data Availability

The original contributions presented in the study are included in the article/Supplementary Material, further inquiries can be directed to the corresponding author/s.
